# Molecular Basis to Integrate Microgravity Signals into the Photoperiodic Flowering Pathway in *Arabidopsis thaliana* under Spaceflight Condition

**DOI:** 10.3390/ijms23010063

**Published:** 2021-12-22

**Authors:** Junyan Xie, Lihua Wang, Huiqiong Zheng

**Affiliations:** Center for Excellence in Molecular Plant Sciences, Chinese Academy of Sciences, Shanghai 200032, China; jyxie@cemps.ac.cn (J.X.); lhwang@cemps.ac.cn (L.W.)

**Keywords:** spaceflight, microgravity, photoperiod, flowering, *Arabidopsis thaliana*

## Abstract

Understanding the effects of spaceflight on plant flowering regulation is important to setup a life support system for long-term human space exploration. However, the way in which plant flowering is affected by spaceflight remains unclear. Here, we present results from our latest space experiments on the Chinese spacelab Tiangong-2, in which Arabidopsis wild-type and transgenic plants *pFT::GFP* germinated and grew as normally as their controls on the ground, but the floral initiation under the long-day condition in space was about 20 days later than their controls on the ground. Time-course series of digital images of *pFT::GFP* plants showed that the expression rhythm of *FT* in space did not change, but the peak appeared later in comparison with those of their controls on the ground. Whole-genome microarray analysis revealed that approximately 16% of Arabidopsis genes at the flowering stage changed their transcript levels under spaceflight conditions in comparison with their controls on the ground. The GO terms were enriched in DEGs with up-regulation of the response to temperature, wounding, and protein stabilization and down-regulation of the function in circadian rhythm, gibberellins, and mRNA processes. *FT* and *SOC1* could act as hubs to integrate spaceflight stress signals into the photoperiodic flowering pathway in Arabidopsis in space.

## 1. Introduction

Long-duration manned space exploration will require close biological life support systems (BLSS) in which humans can live while reducing the minimum amount of water, oxygen, and nutrients to be transported and optimizing the recycling of reusable waste [1,2]. Such a system will require an edible plant to be successfully cultivated long term in the environmental conditions expected to be achieved during spaceflight and on arrival at nearby objects [3]. Spaceflight conditions, including microgravity or low gravity, radiation, temperature, air and soil composition constraints, etc., greatly affect plant survival and adaption. Moreover, the volume and energy supply in the isolation chambers in space used to cultivate plants are very limited. Thus, to construct some higher resource-use efficiency with high yield plants is necessary for plant-based BLSS.

Flowering time is an important trait in breeding for crop yield and is very sensitive to environment stresses, such as drought, cold, salt, and nutrients [4,5]. As a compensatory mechanism, flowering time as well as seed production are often changed in stressful environments [6,7]. In space, the completion of the seed-to-seed cycle is further constrained due to the presence of factors such as microgravity, which could trigger multiple stressors to affect absorption, transport, and the distribution of water and nutrients in plants, as well as transpiration rates [2,8,9]. The attempts of several pioneering space experiments to grow plants through a complete life cycle were unsuccessful because plants frequently delayed development or died in the transition from the vegetative to reproductive stages [10,11,12,13]. The first plant that produced new seeds in space was Arabidopsis [14]. With the improvement in cultivation chambers, serial space experiments successfully completed a full life cycle with several plants, including Arabidopsis, *Brassica rapa*, pea, and wheat [15,16,17,18,19]. However, plants often developed reduced numbers of flowers, changed inflorescence structures, and developed smaller and more lightweight seeds, with storage reserves changed in comparison with ground control plants in these space experiments [20]. This is a risk for the achievement of resource-use efficiency in plant-based BLSS. The increase in plant reproduction in space has begun to attract more attention because of the opportunity to conduct long-term experiments in space provided by space stations and the increasing demand for higher efficiency BLSS for long-duration spaceflights.

One of the most important processes for plant reproductive development is flowering time, which is regulated by day length (photoperiod pathway), prolonged periods of cold (vernalization pathway), the autonomous pathway, and the gibberellic acid pathway [21]. These different pathways converge to regulate the expression of the floral integration genes *SUPPRESSOR OF OVEREXPRESSION OF CO1* (*SOC1*) and *FLOWERING LOCUS T* (*FT*) that act in concert to initiate flowering. Photoperiod and temperature information in Arabidopsis is processed through a circadian clock-dependent mechanism to induce the expression of *FT*, in long-day (LD) [22]. The induction of *FT* occurs in leaves, in which the FT protein is transferred to the shoot apical meristem to promote flower formation together with SOC1, which is locally produced in response to FT accumulation [23]. This mechanism of photoperiod-controlled flowering originally characterized in Arabidopsis is highly conserved in angiosperms (including major crops, such as rice, wheat, barley, and potato). In addition, Arabidopsis has played a very important role in understanding plant adaption to spaceflight, especially plant growth and tropisms [2,24,25].

Our previous experiments studied the flowering of Chinese cabbage and Arabidopsis plants in space on board the Chinese recoverable satellites SJ-8 and SJ-10. In these two flight experiments, plants had fully developed rosettes at the time of launch and initiated flowering shoots while in orbit [26]. The aim of the present work was to examine how photoperiod conditions affect the flowering time of plants grown under microgravity in space on board the Chinese spacelab TG-2 from seeds to seeds. The photoperiod response depends on the induction of *FT* [22]. To understand the role of how *FT* is induced in response to the photoperiod response in space, we generated a *pFT::GFP* transgenic plant (*pFT::GFP*) construction to study the regulation of *FT* assessed by GFP fluorescence. The WT and *ft-10* mutant plants were used as controls. In addition, the altered expression of genes in the flowering signaling pathway of plants grown under spaceflight conditions is also analyzed.

## 2. Results

### 2.1. Effects of Microgravity on Flowering of Plants under Different Photoperiodic Conditions

**Figure 1:**
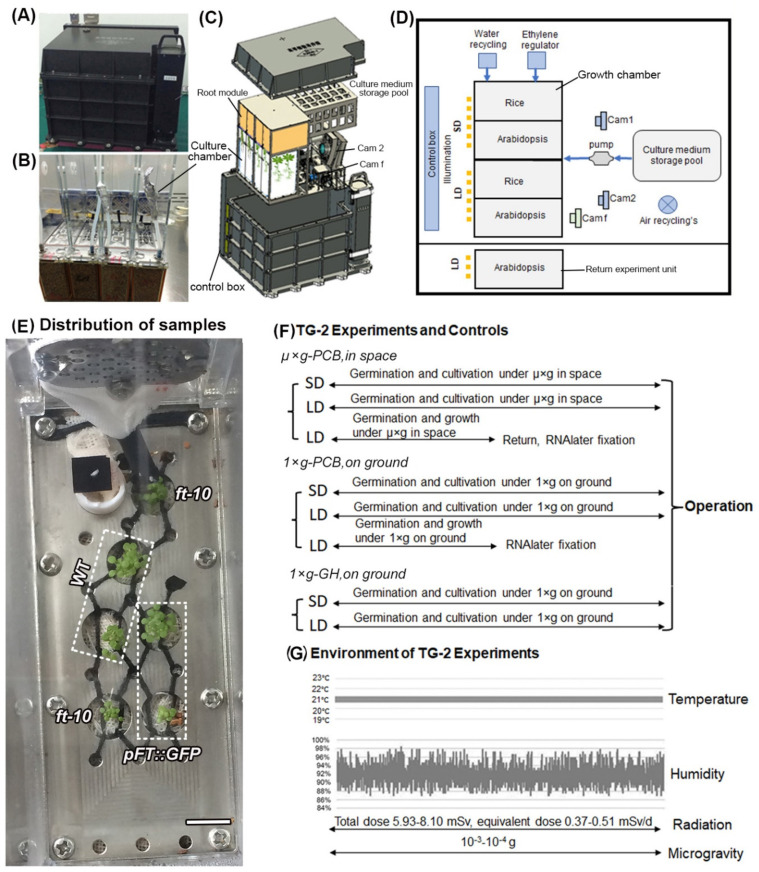
The plant culture box (PCB) on board the Chinese spacelab TG-2 and experimental design. (**A**) The outside view of the PCB, the dimensions are 300 × 300 × 400 mm^3^. (**B**) Culture chambers. (**C**) The assembly of the inside view of the PCB with cover, showing growth chamber, culture medium pool, and three digital cameras. (**D**) Diagram of the composition inside the PCB, showing water recycling, ethylene regulator, nutrient delivery, illumination system, image acquirement subsystem including two cameras for acquisitioning visible images (Cam 1 and Cam 2) and one camera for fluorescence images acquisition (Cam f). (**E**) An example image to show distribution of samples on the installation panel of culture chambers. Scale bar = 10 mm. (**F**) Schematic representation of the scenarios used in space experiments in PCB under microgravity on board the Chinese spacelab TG-2 (µ× *g*-PCB) and their corresponding controls at the 1× *g* gravity condition on the ground in PCB (1× *g*-PCB) or in a greenhouse (1× *g*-GH). The experiments comprised two photoperiod conditions: Long-day (LD, 16 h light/8 h dark) and short-day (SD, 8 h light/16 h dark). (**G**) Temperature, humidity, radiation, and microgravity conditions are indicated for each experiment.

Arabidopsis wild-type (WT), *pFT::GFP* transgenic plants (*pFT::GFP*), and *ft-10* mutant plants were grown in culture chambers (CCs), which were installed in plant culture boxes (PCBs) under microgravity (µ× *g*) on board the Chinese spacelab TG-2 (µ× *g*-PCB) and under normal gravity (1× *g*) on the ground either in PCB (1× *g*-PCB) or in a greenhouse (GH) (1× *g*-GH), as described above (Figure 1).

**Figure 2:**
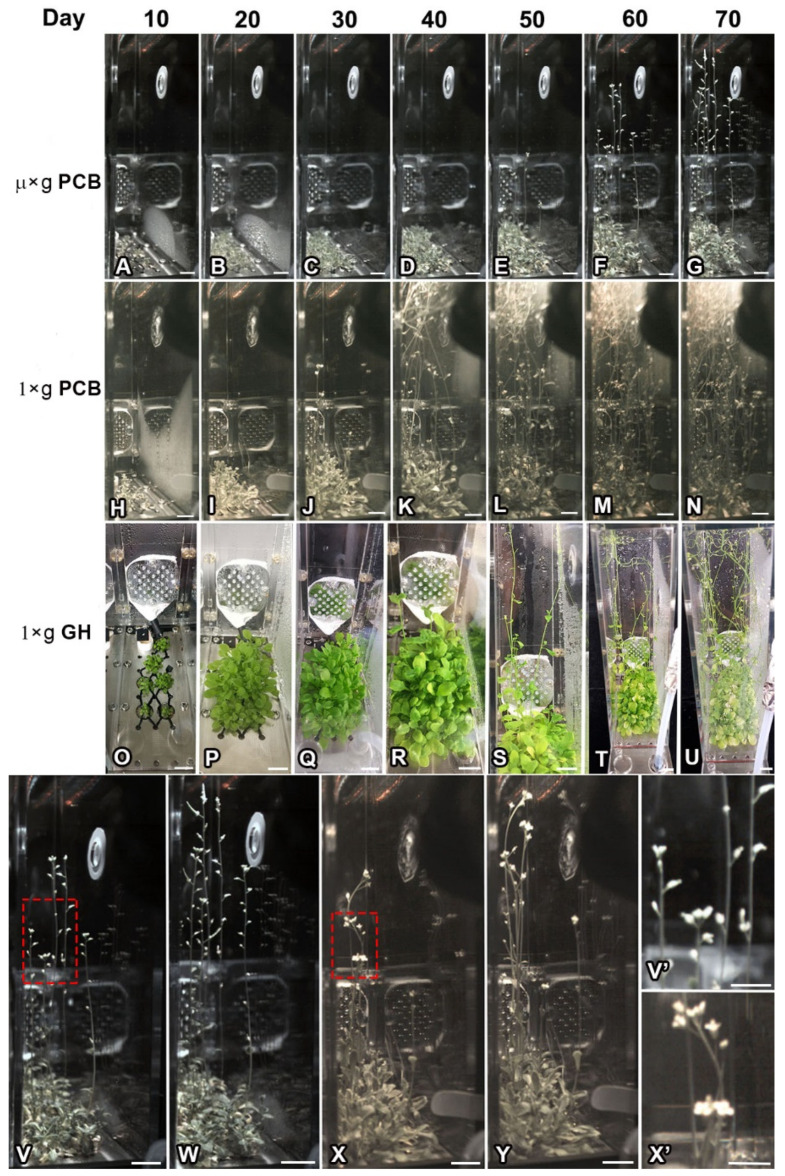
Response of Arabidopsis plants growth under the long-day (LD) condition under microgravity in space in comparison with that of the controls on ground. (**A**–**U**) Example images of plants were recorded at days 10, 20, 30, 40, 50, 60, and 70 after sowing in plant culture box (PCB) in space (**A**–**G**) and on ground either in PCB (**H**–**N**) or in greenhouse (GH) (**O**–**U**), respectively. (**V**–**Y**) Magnified views of the plants in PCB at day 60 and day 70 in space (**V**,**W**) and on day 31 and day 32 on ground (**X**,**Y**). (**V’**,**X’**) Enlarged images of the red framed area in (**V**) and (**X**), respectively. Note that flowers could not fully open in space (**V’**) in comparison with that of the ground controls (**X’**). µ× *g*-PCB, in PCB under microgravity in space. 1× *g*-PCB, in PCB under the normal gravity (1× *g*) on ground. 1× *g*-GH, in greenhouse under the normal gravity on ground. Scale bar = 10 mm in (**A**–**Y**); 5 mm in (**V’**,**X’**).

**Figure 3:**
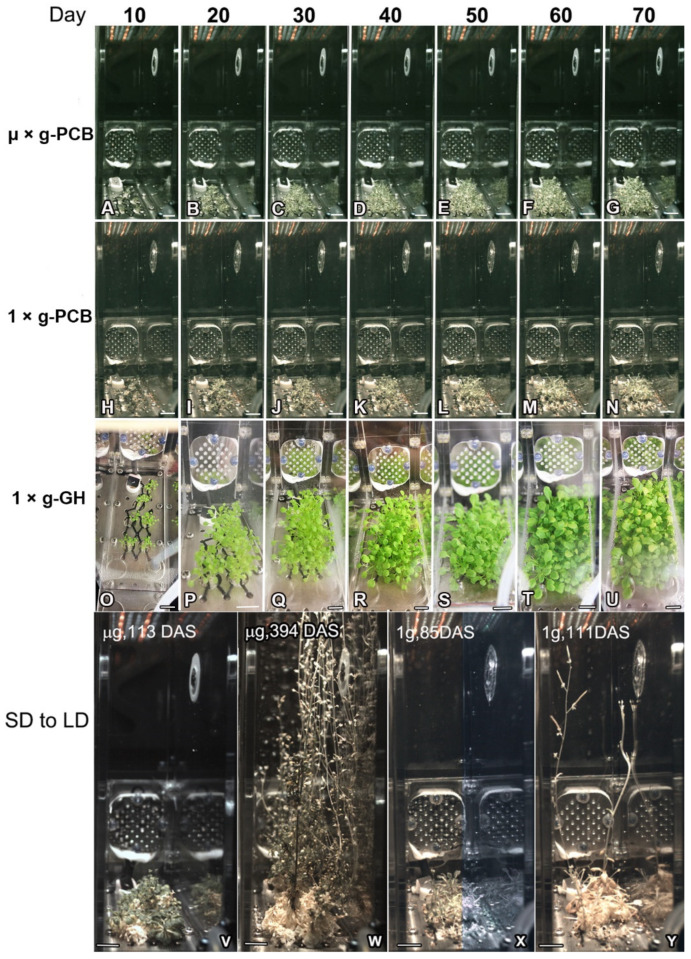
Time-course series of digital images showing the growth of Arabidopsis plants under short-day (SD) conditions in space and on ground. (**A**–**U**) Example images of plants under the SD were recorded at days 10, 20, 30, 40, 50, 60, and 70 after sowing in PCB in space (**A**–**G**) and on ground in either PCB (**H**–**N**) or greenhouse (**Q**–**U**). (**V**,**W**) Example images of the plants in PCB in space at the stage of the first bolting (113 DAS) and seed maturity with senescence complete (394 DAS) after being transferred from the SD to the LD condition. (**X**,**Y**) Example images of the plants in PCB on ground at the stage of the first bolting (85 DAS) and seed maturity with senescence complete (111 DAS) after being transferred from the SD to the LD condition. µ× *g*-PCB, Plants in PCB under microgravity in space. 1× *g*-PCB, Plants in PCB under the normal gravity (1× *g*) on ground. 1× *g*-GH, Plants in greenhouse under the normal gravity on ground. Scale bar = 10 mm.

Downloaded images revealed that seeds of WT, *pFT::GFP* transgenic plants and the *ft-10* mutant in CCs under both the LD and SD conditions in µ× *g*-PCB started to germinate from 3 days after being sown (DAS). Seeds in space germinated well irrespective of photoperiodic conditions (Figure 2 and Figure 3). After germination, WT, *pFT::GFP,* and *ft-10* plants developed rosette leaves in either LD or SD in µ× *g*-PCB. On 10 DAS, expanded rosette leaves spread upward away from the plate under LD in CCs in µ× *g*-PCB and on the ground either in 1× *g*-PCB or 1× *g*-GH (Figure 2A,H,O), while those with elongated hypocotyls and smaller cotyledon under SD were observed in the three growth conditions (Figure 3A,H,O and Figure S1).

**Figure 4:**
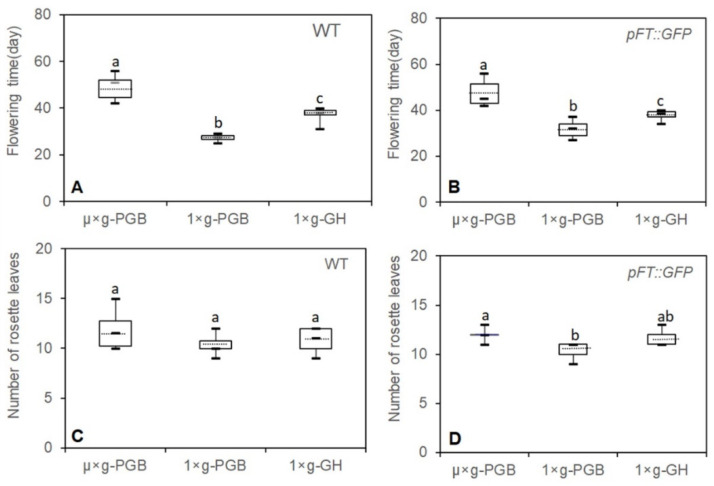
Flowering time of plants grown under long-day (LD) conditions in space and on ground was recorded. (**A**,**B**) Flowering time of Arabidopsis wild-type (WT) and transgenic plants (*pFT::GFP*) grown in culture chambers under microgravity (µ× *g*) in the space plant culture boxes (µ× *g*-PCB) and under the normal gravity (1× *g*) in the ground PCB (1× *g*-PCB) or in GH (1× *g*-GH) was recorded. (**C**,**D**) Total leaf number was counted when floral buds became visible. Each box plot shows 1.5 × interquartile range of the data points, third quartile, median and first quartile value. Dotted lines show mean value (*n* = 6–10). Different letters indicate statistical significance by Student’s *t*-test (*p* < 0.01).

WT and *pFT::GFP* transgenic plants grown under LD in µ× *g*-PCB flowered later than those under the same photoperiodic condition on the ground in either 1× *g*-PCB or 1× *g*-GH (Figure 2E,J,Q and Figure 4). The first bolting (start of peduncle growth) took place on 42 DAS (median 48 DAS for WT; 47 DAS for *pFT::GFP*) in µ× *g*-PCB, and on 25 DAS (median 27 DAS) for WT and 27 DAS (median 32 DAS) for *pFT::GFP* on the ground in 1× *g*-PCB, on 31 DAS (median 37 DAS) for WT and 34 DAS (median 37 DAS) for *pFT::GFP* in 1× *g*-GH (Figure 2 and Figure 4A,B). In addition, the spaceflight plants (both WT and *pFT::GFP*) also had approximately two more rosette leaves at flowering than their control plants on the ground (Figure 4C,D). However, statistical analysis showed that there was no significant difference for WT among the three growth conditions and for *pFT::GFP* transgenic plants between µ× *g*-PCB and 1× *g*-GH conditions, except for a significant difference between µ× *g*-PCB and 1× *g*-PCB conditions (Figure 4C,D).

The plants grown under SD in µ× *g*-PCB did not flower until 70 DAS, nor as well as those on the ground (both 1× *g*-PCB and 1× *g*-GH) did (Figure 3 and Figure S2). To further determine the influence of microgravity on daylength-regulation flowering, we transferred these plants, both in spaceflight and on the ground, from SD to the LD condition. The results showed that WT and *pFT::GFP* plants in µ× *g*-PCB started bolting at day 43 (at 113 DAS) after being transferred from SD to LD (Figure 3V), while those in 1× *g*-PCB began bolting at day 15 (75 DAS) after being transferred to LD (Figure 3X). Finally, plants reached the stages of seed maturity with complete senescence on day 324 in µ× *g*-PCB in space (Figure 3W) and on day 41 in 1× *g*-PCB on the ground (Figure 3Y) after being transferred from the SD to the LD.

### 2.2. Application of GFP Technique for Detection of FT Expression in Space

**Figure 5:**
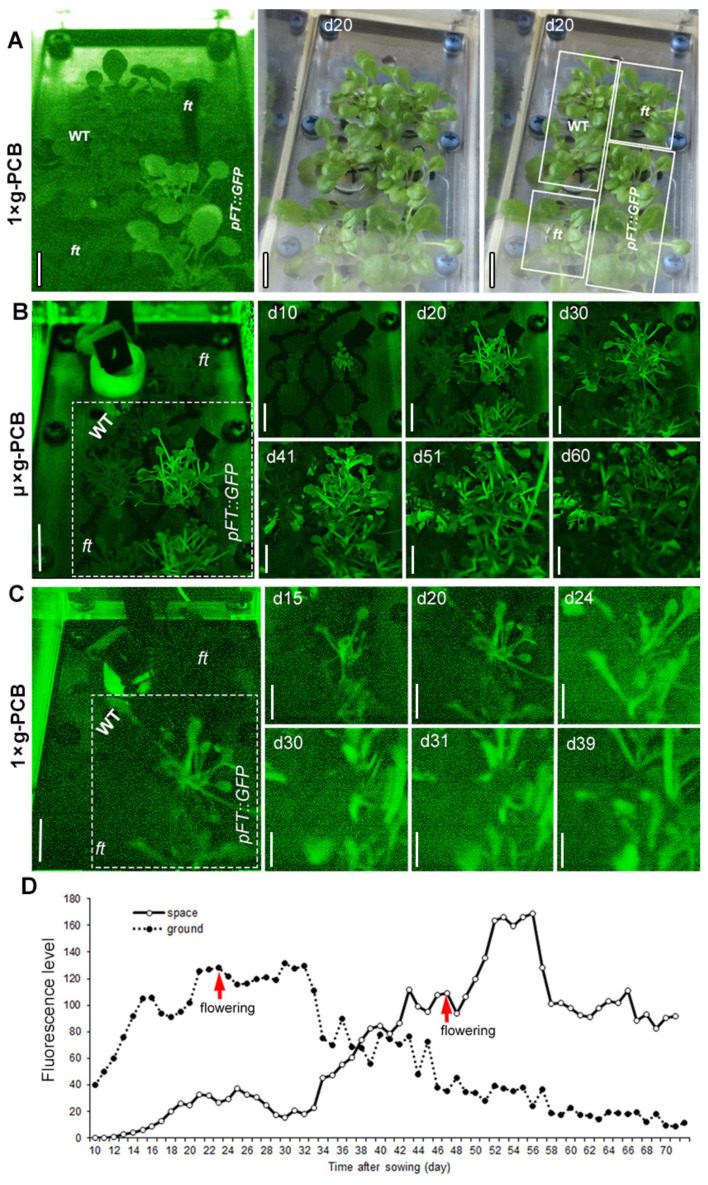
Expression pattern of *pFT::GFP* in the leaves of Arabidopsis plants grown under LD in space and on ground. (**A**) An example images of *pFT::GFP* seedlings grown in a culture chamber (CC) in plant culture box (PCB) under LD on ground to indicate the position of plants in the CC. Intense GFP expression can be seen in the 20-day-old transgenic plants (*pFT::GFP*) in comparison with that of wild-type (WT) and *ft* mutant plants at the same age. The fluorescence image can distinguish *pFT::GFP* plants expressing GFP from WT and *ft* plants, which did not express GFP. The non-expressing WT and *ft* plants are faintly visible because of the fluorescence caused by excitation of chlorophyll. In these *pFT::GFP* plants, the normal fluorescence of the leaves is overpowered by the green fluorescence of the GFP. The two right images show the same CC photographed in white light. (**B**,**C**) Example images of plants were taken autonomously by the camera in the PCB (Cam f in Figure 1C) under LD in space (**B**) and on ground (**C**). All images were remotely downloaded as image data packets. The large image to the left shows the full camera view, the smaller serial images were cropped to focus on *pFT::GFP*. µ× *g*-PCB, Plants in PCB under microgravity in space. 1× *g*-PCB, Plants in PCB under the normal gravity (1× *g*) on ground. Scale bar = 10 mm. (**D**) Quantification of *pFT*::GFP signal in the leaves of plants in space and on ground.

The expression pattern of *pFT::GFP* in the leaves of Arabidopsis plants grown under LD in µ× *g*-PCB and 1× *g*-PCB was monitored by the GFP Imaging System (GIS) (Figure 5A). In the leaves of plants grown under LD in 1× *g*-PCB on the ground, the expression of *GFP* rapidly increased from 10 DAS and reached peak levels at approximately 22 DAS (Figure 5C,D). In contrast, *GFP* expression in the leaves of plants under the same condition in µ× *g*-PCB in space remained at a low level during the first 15 DAS, and reached a higher level at about 43 DAS, then continued to rise to peak levels at 50–55 DAS (Figure 5B,D). The peak level of *GFP* expression in the µ× *g*-PCB plants was apparently higher than their control on the ground (Figure 5D).

**Figure 6:**
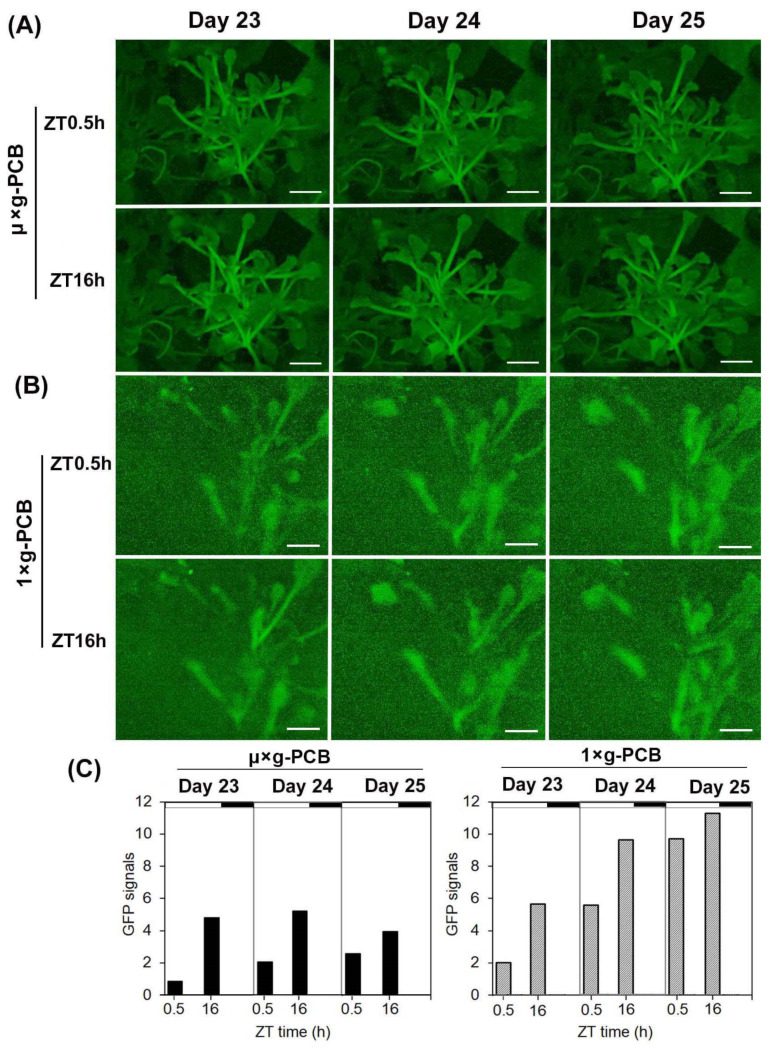
Time-course series of digital GFP images showing morning and evening *GFP* expression level under LD condition in space. (**A**,**B**) *GFP* expression profiles in *pFT::GFP* transgenic plants in morning (ZT0.5) and evening (ZT16) under the LD condition in space (**A**) and on ground at 23, 24, and 25 days after sowing (DAS). µ× *g*-PCB, Plants in PCB under microgravity in space. 1× *g*-PCB, Plants in PCB under the normal gravity (1× *g*) on ground. Bars = 5 mm. (**C**) Quantification of *GFP* signal in the leaves of plants in space and on ground.

The induction of *FT* expression with a diurnal rhythm was considered the most effective to promote Arabidopsis flowering under LD conditions [27]. To examine whether this dynamic of *FT* expression also encodes LD information in spaceflight plants, we compared the level of *pFT::GFP* expression in leaves of plants in µ× *g*-PCB in space at Zeigeber time 0.5 h (ZT: time after light on set) and ZT16 h, respectively, with that in plants in 1× *g*-PCB on the ground (Figure 6). The expression level of *GFP* in both spaceflight plants and the ground controls increased more at ZT 16 than that at ZT 0.5, but the amplitude of the enhanced daily level in spaceflight plants was smaller than that in the ground controls (Figure 6C). These results indicate that the diurnal rhythm pattern of *FT* expression under LD in space remained the same as their ground controls, but the expression level increased in smaller amplifications.

### 2.3. Altered Expression of Genes in Arabidopsis Leaves at Flowering Stage in Space

**Figure 7:**
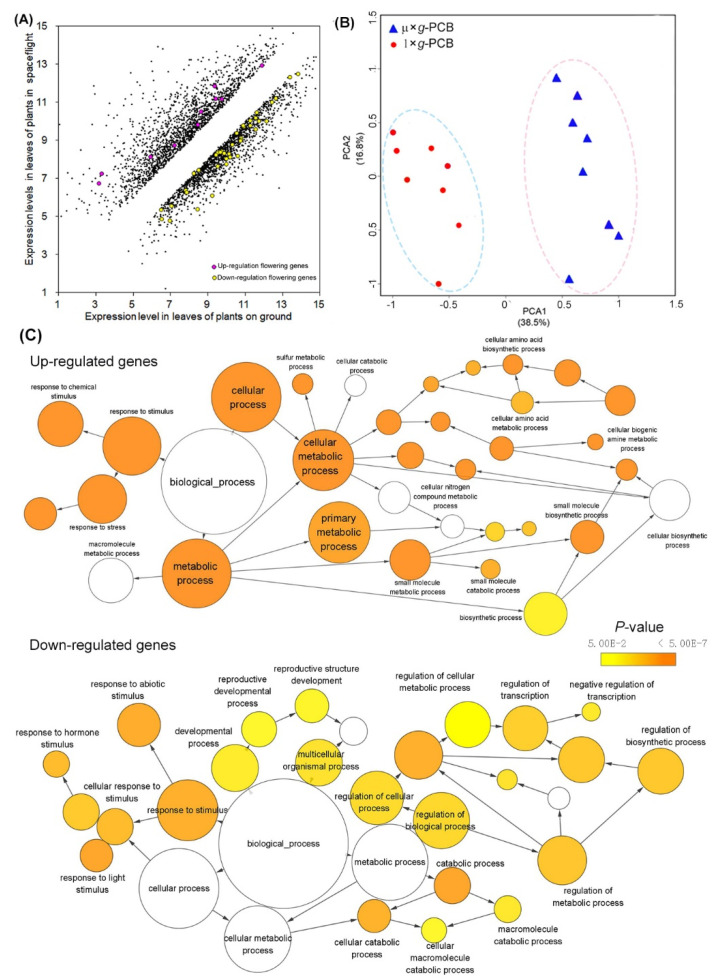
Changes in the expression of genes occurring in spaceflight plant leaves. (**A**) Scatter plot of changed expression level of genes in 48-d-old spaceflight plants in µ× *g*-PCB in comparison with those of ground control in 1× *g*-PCB at the same developmental stage. (**B**) Principal component analysis (PCA) demonstrates a strong difference between the space flight (µ× *g*-PCB) and the ground control (1× *g*-PCB) sample transcription. (**C**) Enriched GO terms in differential expression genes (DEGs) in A. The networks graphs show BiNGO visualization of the overrepresented GO terms for up-regulated and down-regulated DEGs, respectively. The same edge degree is shown on the two graphs. Uncolored nodes are not overrepresented, they may be the parents of overrepresented terms. Colored nodes represent GO terms that are significantly overrepresented (Benjamini and Hochberg corrected *p*-value ˂ 0.05), with the shade indicating significance as shown in the color bar.

**Figure 8:**
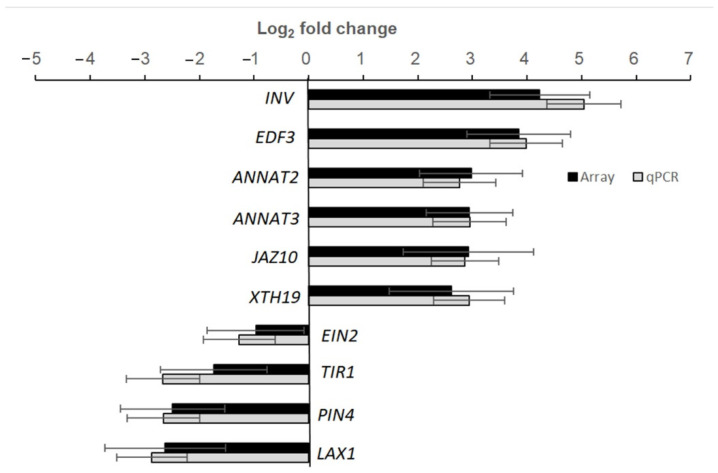
Relative transcript abundance changes in response to microgravity as compared to the ground control were analyzed by microarray and real-time qPCR using SYBR Green for detection. Microarray data for *INV* (AT3G17130), *EDF3* (AT3G25730), *ANNAT2* (AT5G65020), *ANNAT3* (AT2G38760), *JAZ10* (AT5G13220), *XTH9* (AT4G30290), *EIN2* (AT5G03280), *TIR1* (AT3G62980), *PIN4* (AT2G01420), and *LAX1* (AT5G01240) for an average of four independent technical replicates. Real-time qPCR with isoform-specific primers for those genes was performed on a fourth independent technical replicate using the same samples as in the microarray analysis above. Error bars indicate the standard deviation of the mean.

To explore the influence of spaceflight on a whole transcription scale, we performed microarray analysis using the leaves of WT plants grown under LD on board TG-2 in µ× *g*-PCB and on the ground in 1× *g*-PCB. The samples were harvested at ZT16 when the expression of *FT* was at the peak and fixed with RNA later. RNA was extracted and the altered expression of the genes in µ× *g*-PCB plants was identified by comparing it with the expression level of the genes in 1× *g*-PCB plants on the ground when controlling the false discovery rate (FDR) at the level of 0.05 using the method of Storey and Tibshirani (2003) [28]. Of the genes that met these criteria, we rank-ordered them by fold change (FC). The genes in which the expression level was changed more than 2 (FC ≥ 2) were selected as differential expression genes (DEG). Figure 7A showed that 3462 genes of µ× *g*-PCB samples exhibited differential expression in comparison with those in 1× *g*-PCB plants on the ground (Table S1). Principal component analysis (PCA) of the samples demonstrated a strong difference between the transcriptomes of samples grown in µ× *g*-PCB in space and in 1× *g*-PCB on the ground (Figure 7B). Compared with those on the ground in 1× *g*-PCB, 1793 genes were up-regulated, while there were 1669 down-regulated genes in µ× *g*-PCB samples (Figure 7A and Table S1). GO term enrichment analysis showed that up-regulated genes are involved in the metabolic process, and the response to stress was enriched (Figure 7C, Table S2a), while down-regulated genes function in response to stimulus, the reproductive developmental process, and the regulation of the metabolic process (Figure 7C, Table S2b). To validate the microarray data, we generated sequence-specific primers and performed real-time qRT-PCR on a third independent replicate. Real-time PCR with isoform-specific primers for plant invertase (*INV*, AT3G17130), ethylene response DNA binding factor 3 (*EDF3*, AT3G25730), annexin 2 (*ANNAT2*, AT5G65020), annexin 3 (*ANNAT3*, AT2G38760), jasmonate-zim-domain protein 10 (*JAZ10*, AT5G13220), xyloglucan endotransglucosylase/hydrolase 19 (*XTH19*, AT4G30290), ETHYLENE INSENSITIVE 2 (*EIN2*, AT5G03280), F-box/RNI-like superfamily protein (*TIR1*, AT3G62980), auxin efflux carrier family protein (*PIN4*, AT2G01420), and auxin transporter-like protein 1 (*LAX1*, AT5G01240) confirmed the relative abundance changes for the transcript levels of these genes in µ× *g*-PCB samples in comparison with those in 1× *g*-PCB samples (Figure 8).

**Figure 9:**
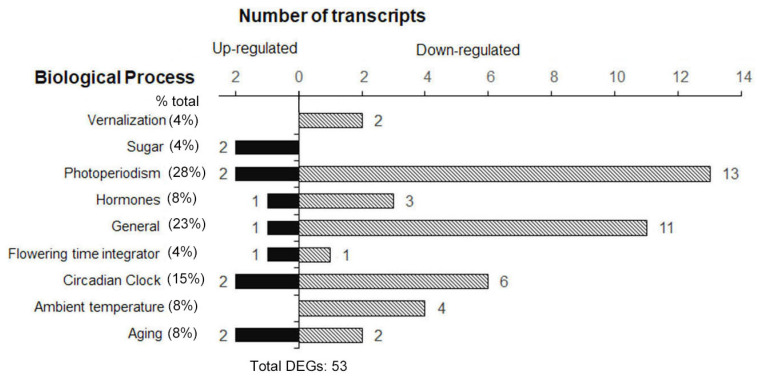
Functional classification of differential expression flowering genes (DEGs) in wild-type plants grown under the LD condition in space in comparison with their controls under the same condition on ground.

**Figure 10:**
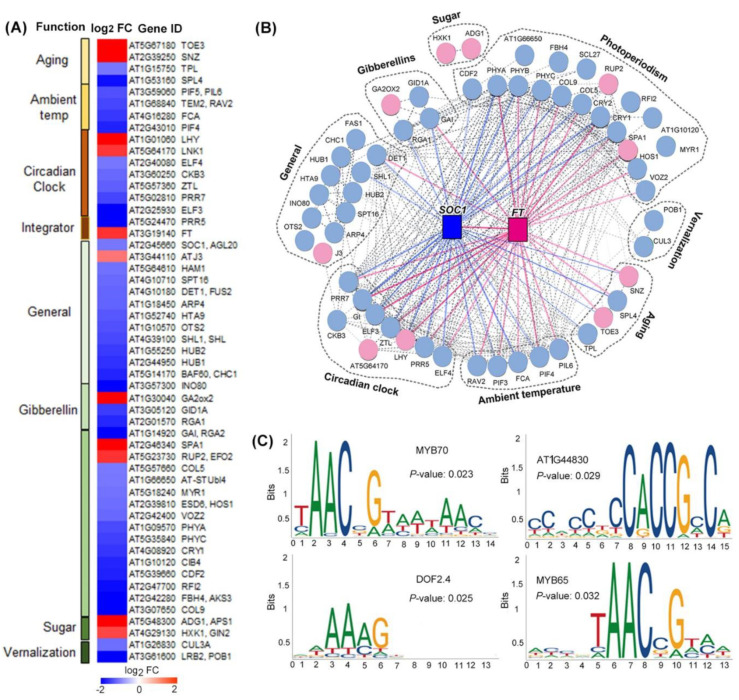
Interaction networks of flowering genes in response to spaceflight conditions. (**A**) Heatmap showing fold changes (FC) in transcript abundance of spaceflight samples in comparison with the ground control (log_2_ FC). (**B**) Diagram of the protein interaction networks of flowering genes, which up-regulated and down-regulated expression levels under spaceflight conditions in comparison with their controls on ground. The up-regulated genes are shown in pink and down-regulated genes are shown in blue. (**C**) Four overrepresented motifs enriched in upstream promoter sequences of genes in flowering function classification in Figure 9, as detected by the plant regulomics (bioinfo.sibs.ac.cn/plant-regulomics, update 9 July 2021). Indicated are the *p*-values representing the statistical significance of the motif.

Based on FLOR-ID [29], 53 flowering genes were identified among the DEGs (Figure 9). We classified this set of 53 transcripts according to their known or predicted function in the flowering regulation pathway. Most of these flowering genes regulated by spaceflight were functionally categorized to be involved in photoperiodism and light perception and signaling (28% of total DEGs in the flowering pathway), 15 of these DEGs function in photoperiodism, 2 genes were up-regulated, while 13 genes showed a decrease in transcript abundance. In the following, DEGs function in general (23%), circadian clock (15%), gibberellins (8%), ambient temperature (8%), aging (8%), vernalization (4%), and sugar (4%) were also observed (Figure 9). The data showed that an overall down-regulation of the light response, gibberellins, circadian clock, and ambient temperature occurred. At the same time, a few genes involved in photoperiodism, the circadian clock, sugar, gibberellins, and aging up-regulated expression levels in response to spaceflight were also observed (Figure 10A,B). In addition, two flowering time integrator genes, *FT* and *SOC1*, which changed expression levels in µ× *g*-PCB in comparison with the controls in 1× *g*-PCB, were also identified (Figure 10B). These results indicate that Arabidopsis plants could develop a mechanism by which spaceflight stresses, including microgravity, are transmitted to signaling pathways to be integrated into flowering regulation pathways.

To test whether there are potential common cis-acting elements among these spaceflight responsive flowering genes, we performed analyses using Plant Regulomics (bioinfo.sibs.ac.cn/plant-regulomics) to find overrepresented motifs in the 1-kb upstream sequence of the overrepresented genes in these 53 flowering genes. Thirty coregulated genes were identified to share four common motifs, of which the occurrence was significantly high as compared to that in random genomic regions (Figure 10C). These include (T/C)AAC (C/G)G, which is identical to MYB70 (AT2G23290) and MYB 65 (AT3G11440). The expression of genes regulated by MYB65 was consistent with roles in GA-mediated processes, with the expression of MYB 33 at the shoot apex coinciding with the onset of flowering [30,31]. C (A/GCCG (C/A)C represents the motif identified by the integrase-type DNA-binding superfamily protein (AT1G44830) encoding a member of the DREB subfamily A-5 of the transcription factor family, and the protein contains one AP2 domain, which plays an important role in plant flowering, seed development, and abiotic stress response [32]. AA (A/C) G is identical to the motif of DOF2.4 (AT2G37590) [33]. This result indicates that common regulators might be involved in the regulation of plant flowering under spaceflight conditions.

## 3. Discussion

Microgravity in spaceflight is considered a special type of stress, which can significantly affect plant growth and development, including plant flowering. In this study, we have performed a seed-to-seed experiment on board the Chinese Spacelab TG-2. Image analyses suggested that *FT* expression in the leaves of plants under spaceflight conditions was apparently changed in comparison with that in the ground controls. Our transcriptional data show that the alteration in responses of plants in space to temperature, circadian clock, and gibberellins could potentially affect their flowering regulation under spaceflight conditions.

The recordings of growth and flowering time under the LD and SD conditions reported here under microgravity are unique, although earlier studies of seed-to-seed growth in *Arabidopsis thaliana* have been performed in space [17,19,34,35]. The problems related to embryo and seed development in space and the lower quality and quantity still remain [19,36,37]. Damage caused during the flowering phase of a plant is irreversible and can cause reproductive failure [6]. However, a few space experiments so far have focused on this key developmental stage. Our study indicated that the initiation of bolting in Arabidopsis plants was delayed under the LD condition on board spaceflight. This result was confirmed by comparing the flowering time of the plants grown under SD firstly then transferring them to the LD condition in space with their ground controls. However, our result is different from a previous report, which suggested that the timing of bolting in Arabidopsis plants tended to be earlier in space on board the international space station (ISS) in comparison with the 1g space control [19]. Different from our experiment, which was performed under the LD and SD conditions, Karahara et al.’s experiment was performed under a continuous light (LL) condition [19]. Arabidopsis plants grown at high densities under LL were reported to have accelerated flowering times in comparison with LD and SD [38]. Plants grown under LL conditions could be affected by a disruption of circadian rhythms without a light–dark cycle [39,40]. We describe information in this study as to the effect of spaceflight factors on flowering time in Arabidopsis in space under two different typical daylength conditions, LD and SD, which are the key issues for vegetable and crop production in a closed cultivation system such as BLSS or a plant factory.

Since the opportunity to conduct experiments in space is very limited, the application of living image in-flight and transgenic plants harboring the target genes is important to enhance scientific productivity by rapid feedback of results during missions and increase the on-board analysis capability. For example, transgenic Arabidopsis plants containing the alcohol dehydrogenase gene promoter linked to the ß-glucuronidase (GUS) reporter gene have been utilized to address various aspects of spaceflight-specific hypoxic stress in shuttle experiments [41,42]. Using the GFP reporter gene system, Ferl and Paul (2015) analyzed the effect of spaceflight on the gravity-sensing auxin gradient of Arabidopsis roots [43]. The present results extend the observation to the expression of the *GFP* gene under *FT* promoter control in Arabidopsis transgenic plants in space. FT has great importance in vegetative and reproductive growth because several flowering pathways, including the long-day photoperiod, vernalization, autonomous promotion, and temperature-dependent pathways, are integrated into the regulation of *FT* expression [23]. According to downloaded images taken by online cameras, our study demonstrated that the expression rhythm of *FT* in Arabidopsis plants grown in space was unchanged, but the peak of *FT* expression appeared later.

Our results indicate that *FT* and *SOC1*, which are in concert to initiate flowering, could act as integration hubs in the regulation pathways of plant flowering in response to spaceflight (Figure 9). Consistent with the expression of *GFP* as described above, *FT* in the leaves of spaceflight plants was also up-regulated at the time of flowering induction and is accompanied by the up-regulated expression of the circadian clock gene LHY and the photoperiodic gene SPA1. SOC1 is regulated by a complex transcriptional regulatory network that allows for the integration of multiple floral regulatory inputs from photoperiods, gibberellins, and FLOWERING LOCUS C [44]. Our result demonstrates that the expression of *SOC1* decreased in leaves under spaceflight conditions accompanied with the down-regulation of key genes involved in the response to the photoperiod, circadian clock, vernalization, ambient temperature, gibberellins, and aging. This indicates that the floral integration genes, *FT* and *SOC1*, could be key genes for Arabidopsis plants to integrate spaceflight-condition stress signaling into flowering regulation pathways.

Space experiments using different space vehicles have been a fascinating tool for understanding the effect of microgravity on plant growth and development. However, most of these experiments have focused on specific phenomena in the short term in space, such as gravitropism and gravimorphogensis, while relatively few studies have aimed to promote plant reproduction in space. After space missions become longer and there are permanently manned stations, the optimization of plant growth and reproduction becomes a requirement to finally set up a plant-basis self-sustained BLSS for long-duration human missions. In the design of plant-based BLSS, the final goal should not be the growth of plants in the short term, but the creation of stable conditions to guarantee plant growth and reproduction to reach the ambitious objective of realizing long-term, self-sustaining space habitats [2]. How to increase the production efficiency of plants will be the most important task in setting up a self-sustaining closed BLSS. Acceleration of flowering time in seed crops, such as rice and wheat, alongside the prevention of bolting in vegetable plants, would increase the input/output ratio of resources obtainable using plants in BLSS. Thus, the control of flowering time by manipulation will be an important biotechnological method to increase the production of BLSS. Our approach introduced new knowledge about the effect of spaceflight factors on plant flowering time. In the future, long-term space experiments from successive generations and systematic analysis of regulatory networks at the molecular level is needed to understand the mechanism of plant flowering control under microgravity (space) and fractional gravity, e.g., on the Moon, where the gravitational acceleration is 1/6 g, or on Mars at 3/8 g.

## 4. Materials and Methods

### 4.1. Plant Materials

*Arabidopsis thaliana* wild-type (WT) ecotype Columbia (Col-0) and the mutant *ft-10* have been previously described [45]. To detect *FT* expression, an 1.5-kb region upstream of the *FT* start codon (*pFT*) was fused to the green fluorescent protein (GFP) coding region, and the *pFT::GFP* transformed plants in the Col-0 background were generated as described previously [13]. Seeds of WT, *pFT::GFP* transgenic plants, and *ft-10* were flown with the Chinese spacelab TG-2.

### 4.2. Spaceflight Procedures

Spaceflight experiments were conducted in a Plant Culture Box (PCB), which provides atmospheric, temperature, and hydration monitoring and control for the plant growth in the long term (the seed-to-seed experiment) as described by Wang et al. (2018) and Wu et al. (2020) [9,46]. Briefly, the PCB consists of five culture chambers (CCs), including two CCs under short-day (SD, 8 h light /16 h dark) and three CCs under long-day (LD, 16 h light/8 h dark) conditions (Figure 1A–D). Two digital cameras for visible image (Cam 1 and Cam2) and one digital camera for fluorescence image (Cam f) acquisition were installed in the PCB, allowing the recording of plants. The photographs of fluorescence images were taken by Cam f in 488 nm blue light through a 510 nm long pass filter with a light device. The cameras in PCB were automatic and preprogrammed and images were downloaded almost in real-time. Healthy seeds of WT, *pFT::GFP,* and *ft-10* were selected and surface-sterilized with 75% (*v*/*v*) ethanol for 1 min, followed by 2% (*v*/*v*) NaClO bleach with 0.01% (*v*/*v*) Triton X-100 detergent for 20 min. After five rinses with sterile water, the seeds were attached to small pieces of cheesecloth (10 × 10 mm, five seeds per piece), then dried under a flow bench. The cheesecloth with seeds was set in the holes on the panel root module of the CC containing commercially available vermiculite (Figure 1B,E). The seeds in the CCs were stored dry for about 20 days prior to the launch of the spacelab TG-2 on 15 September 2016. The experiments were controlled remotely from the Beijing space center and initiated via hydration of the seeds on 23 September 2016. Germinated plants were grown at 21 ± 0.1 °C under the light of white/red (2:1) LEDs at 120 μmol m^−2^ s^−1^. Relative humidity was maintained at 88–98% (Figure 1F). The ethylene concentration in the growth chamber was removed as described [26]. After being grown for 48 days on board TG-2, one of the LD CCs with Arabidopsis plants was taken out from the PCB by an astronaut and fixed with the RNAlater solution for transcriptomic analysis on the ground. This CC was returned to the Earth with the Chinese spacecraft SZ-11 on 18 November 2016 while the other four CCs in the main box remained on board the TG-2 until the plants bore seeds.

Ground control experiments were carried out in parallel with CCs in an engineering reproduction of the spaceflight PCB, or in the greenhouse in Shanghai (Figure 1E). All dimensions, light, temperature, humidity, and control set points were the same as for the spaceflight experiment, but ten days after the spaceflight experiment.

### 4.3. Measurement of Flowering Time and Fluorescence Intensity

Seeds were sown in PCB under LD and SD as described above. The number of rosette leaves and the flowering time of plants were monitored in space and on the ground in PCB with the CCD cameras using the tracer program. The image sampling rate was one image every 2 h during the light period. The flowering time was measured by scoring both the number of rosette leaves on the main stem after bolting according to downline images and flowering time after sowing [41]. The images of plants at the stages of germination to bolting were analyzed. Statistical tests were performed using SAS version 9.4 software (SAS Institute Inc., Cary, NC, USA).

Quantification of the fluorescence intensities of the downloaded GFP images of *pFT::GFP* plants was performed by Image J software (version 1.51j8, National Institutes of Health, Bethesda, MD, USA) [47].

### 4.4. RNA Extraction and Microarray Analysis

Total RNA was extracted from the rosette leaves of space plants and their ground controls in PCB, then purified using the miRNeasy Mini Kit (Cat#217004, QIAGEN, GmBH, Dusseldorf, Germany) following the manufacturer’s instructions, and checked for a RIN number to inspect RNA integration by an Agilent Bioanalyzer 2100 (Agilent technologies, Santa Clara, CA, USA). RNA was amplified, labeled, and purified using the GeneChip 3′ IVT PLUS Reagent Kit (Cat#902416, Affymetrix, Santa Clara, CA, USA) following the manufacturer’s instructions to obtain biotin-labeled cRNA. The array hybridization and wash were performed using the GeneChip^®^ Hybridization, Wash and Stain Kit (Cat#900720, Affymetrix, Santa Clara, CA, USA) in Hybridization Oven 645 (Cat#00-0331-220V, Affymetrix, Santa Clara, CA, USA) and Fluidics Station 450 (Cat#00-0079, Affymetrix, Santa Clara, CA, USA) following the manufacturer’s instructions.

### 4.5. Analysis of Microarray Data

Slides were scanned by the GeneChip^®^ Scanner 3000 (Cat#00-00212, Affymetrix, Santa Clara, CA, USA) and Command Console Software 4.0 (Affymetrix, Santa Clara, CA, USA) with default settings. Raw data were normalized by the MAS 5.0 algorithm and Affymetrix packages in R. Probe sets with signal values lower than the detectable range were adjusted to 75 and probe sets with the values of 75 for all conditions were removed from the subsequent analysis. The averages of normalized ratios are calculated by dividing the average of the normalized signal channel intensity by the average of the normalized control channel intensity. The standard deviation of the space samples (two biological replicates, each biological replicate had three repeats) was employed to identify genes of significant changes relative to the ground controls (*p* value < 0.05). Only genes that showed transcript level changes of at least two-fold in comparison with the ground control and with the same tendency in both biological replicates were considered as relevant for microgravity. Gene Ontology (GO) overrepresentation was performed using PANTHER (Fisher’s Exact type with False Discovery Rate correction) (http://www.pantherdb.org, accessed on 9 July 2021, 16.0 released) [48]. For motif enrichment, motifscan was used to determine whether the occurrence of a given motif in input genes was significantly high as compared to that in random regions [49,50]. For the analysis of the FT and SOC1 protein interaction network in response to spaceflight conditions, data of different expression proteins were used as queries against the string database (http://strigdb.org, accessed on 9 July 2021) to identify protein–protein interactions. Scores were downloaded and exported to Cytoscape (version 3.8.0, San Diego, CA, USA) for network diagrams.

### 4.6. Real-Time qRT-PCR

Total RNA was extracted from leaves of the space samples and the ground controls as described. The genes and their q RT-PCR primers are presented in Table S3. The Arabidopsis ACTIN gene was used as the loading control for all real-time qRT-PCRs. At least four technical replicates of each biological replicate were used for real-time qRT-PCR analysis.

## Data Availability

Not applicable.

## Supplementary Materials

The following are available online at https://www.mdpi.com/article/10.3390/ijms23010063/s1.
