# Supplementary material for: Molecular Basis to Integrate Microgravity Signals into the Photoperiodic Flowering Pathway in Arabidopsis thaliana under Spaceflight Condition
Source: Int J Mol Sci. 2021 Dec 22;23(1):63. doi: 10.3390/ijms23010063 (PMC8744661; doi:10.3390/ijms23010063)
Supplement: Supplementary file 1 [file ijms-23-00063-s001.zip › Supplementary Figures S1 and S2.pdf]

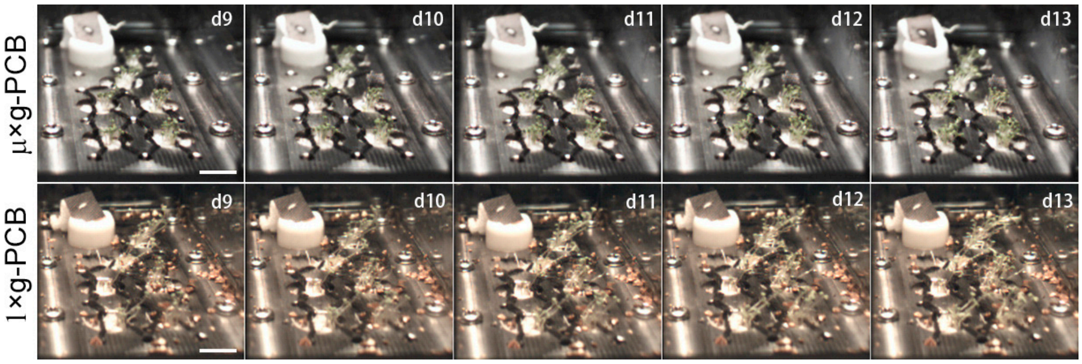

Supplementary Figure S1. Time-course series of images showing of Arabidopsis plants under short-day in space and on ground in plant culture box (PCB). The example images showing seedling under short-day condition from 9-13 day after sowing. Note the plants under SD present elongated hypocotyls and cotyledons smaller than those plants that grow with long-day light conditions. Scan bar=10 mm.

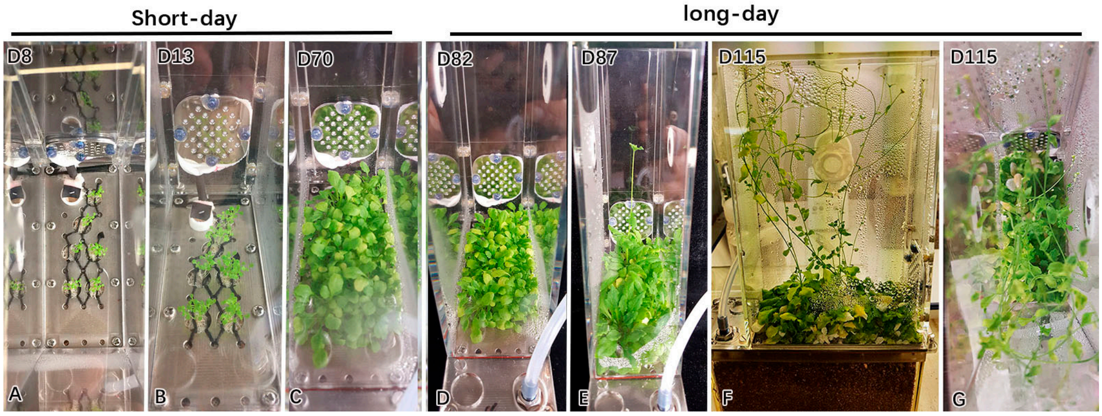

Supplementary Figure S2. Time-course series of images showing of Arabidopsis plants on ground in greenhouse. The plants were firstly grown under SD condition for 70 days (A-C), then transferred to LD condition. (D) Plants started to bolting at 87 days after sowing (day 17 after transferred from the SD to the long-day condition). (F and G) Plants started to mature with senescence around day 115 after sowing (day 45 after transferred from the SD to the LD condition).
