# Supplementary material for: Molecular Basis to Integrate Microgravity Signals into the Photoperiodic Flowering Pathway in Arabidopsis thaliana under Spaceflight Condition
Source: Int J Mol Sci. 2021 Dec 22;23(1):63. doi: 10.3390/ijms23010063 (PMC8744661; doi:10.3390/ijms23010063)
Supplement: Supplementary file 1 [file ijms-23-00063-s001.zip › Supplementary Table S3.pdf]

Table S3. Oligonucleotide primers used in this study

Description	Primer name	Sequence (5' to 3')
Primers of *ANNAT2*	*ANNAT2*-F<br>*ANNAT2*-R	CAACACTCAACCACTACAACAACGA<br>TCGTCCGACTCTTCCTTCAAG
Primers of *JAZ10*	*JAZ10*-F<br>*JAZ10*-R	CCGACCACTCTAAGACCAAAGC<br>TGCGATGGGAAGATCTCCTT
Primers of *EIN2*	*EIN2*-F<br>*EIN2*-R	CAACCGCCTACAGGGAGTGAT<br>GGCAAAAGCACGGTGTCATT
Primers of *LAX1*	*LAX1*-F<br>*LAX1*-R	ATCCCTGCCTTAGCCCACAT<br>GCTAGGGATAAAAAACGGTGGTT
Primers of *TIR1*	*TIR1*-F<br>*TIR1*-R	GCTTCTTTGTCAATGCCCTAAAC<br>GCATCCTCGATGTAGTCAAGCA
Primers of *XTH19*	*XTH19*-F<br>*XTH19*-R	TGCTGGAACCGTCACAACA<br>TCGAAATCAATCTCATCCCATGT
Primers of *EDF 3*	*EDF3*-F<br>*EDF3*-R	GAAAACGGTAACGCCAAGTGA<br>CCGCTTGGTGTTTTGGTATAACT
Primers of plant invertase	*AT3G17130*-F<br>*AT3G17130*-R	CCGACGACCTTATCGACAAAA<br>AGGCTTCACATAGGTCGCAGAA
Primers of *PIN4*	*PIN4*-F<br>*PIN4*-R	TTCTCCGTATAGCCATCGTTCA<br>GCAAACACAAACGGAACTATTCC
Primers of *ANNAT3*	*ANNAT3*-F<br>*ANNAT3*-R	TGATACTCCTGAGAAACACTTTGCA<br>TCATCTGTTCCAAAACCCTCAA
Primers of Actin	*Actin*-F<br>*Actin*-R	TACAGTGTCTGGATCGGTGGTT<br>CGGCCTTGGAGATCCACAT
